# Electrohydraulic lithotripsy with direct peroral cholangioscopy via double-balloon enteroscopy and endoscopic retrograde cholangiopancreatography in a liver transplant recipient

**DOI:** 10.1055/a-2155-4207

**Published:** 2023-09-15

**Authors:** Nicoletta Nandi, Gian Eugenio Tontini, Lucia Scaramella, Andrea Sorge, Tommaso Pessarelli, Matilde Topa, Luca Elli

**Affiliations:** 1 Gastroenterology and Endoscopy Unit, Fondazione IRCCS Caʼ Granda Ospedale Maggiore Policlinico, Milano, Italy; 2 Department of Pathophysiology and Transplantation, Università degli Studi di Milano, Milano, Italy

We present a case of a 36-year-old liver-retransplant recipient with a Roux-en-Y biliary-enteric anastomosis, admitted for cholangitis due to a large bile stone and successfully treated by electrohydraulic lithotripsy (EHL) with direct peroral cholangioscopy (DPCS) through double-balloon enteroscopy-assisted endoscopic retrograde cholangiopancreatography (DBE-ERCP).

The procedure was scheduled after performing a magnetic resonance cholangiopancreatography that showed a 29 × 16-mm stone in the pre-anastomotic duct ( Fig. 1 ). On endoscopy, the bile stone could be seen throughout the anastomosis and showed no signs of stricture. After bile duct canulation and confirmation of stone’s dimension on cholangiography, endoscopic balloon dilation was performed, followed by an unsuccessful attempt to break the stone with a basket ( Fig. 2 ). It was therefore decided to proceed with EHL without the assistance of a cholangioscope given the good visibility of the stone and the possibility to use the enteroscope to enter the dilated bile duct ( Video 1 ). Two EHL probes were required as the first one quickly exhausted, possibly due to its kinking for the DBE loops. Thus, the second one was inserted in an injection needle sheath as protection, resulting in an equally effective probe for a lower power and pulses setting. Complete stone fragment removal was achieved using a basket and a retrieval balloon. No adverse events occurred; the patient was discharged 3 days later.

**Fig. 1 a:**
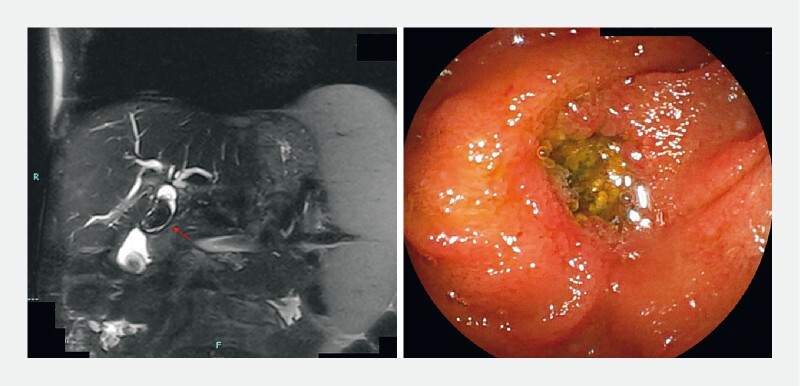
The magnetic resonance cholangiopancreatography shows a dilated main bile duct (17 mm) with a large stone of 29 × 16 mm (red arrow) and a smaller one of 7 mm in the distal hepatic duct, almost at the level of the anastomosis, and bilateral dilation of the intrahepatic ducts. **b** The bile stone is visible through the hepaticojejunal anastomosis before dilation.

**Fig. 2:**
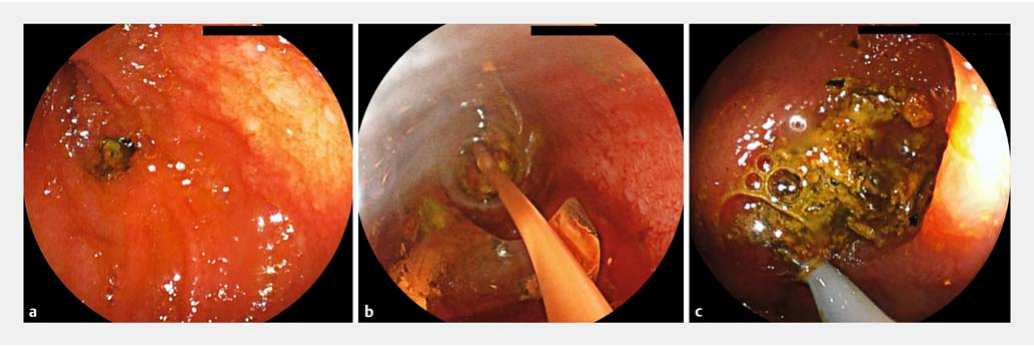
The figure shows the first steps of the procedure before proceeding with electrohydraulic lithotripsy. **a** The hepaticojejunal anastomosis is reached through the afferent limb; the bile stone is wedged on the anastomosis. **b** Endoscopic balloon dilation to 10 mm using a balloon dilation catheter (inflatable up to 3 diameters) under endoscopic and fluoroscopic guidance. **c** Unsuccessful attempt to break the bile stone with a 4-wire basket.

**Video 1** Electrohydraulic lithotripsy performed during double-balloon enteroscopy-assisted endoscopic retrograde cholangiopancreatography under direct endoscopic vision of the enteroscope. This was possible due to good visibility of the bile stone throughout the procedure and the possibility to enter and proceed safely into the dilated hepatic duct with the enteroscope.


DBE-ERCP is an effective technique to treat several biliary conditions in patients with surgical altered anatomy 1 . Successful treatment of complex lithiasis using EHL under DPCS has been described both in normal and altered gastrointestinal anatomy after failure of traditional techniques 2 3 4 5 .

To the best of our knowledge, this is the first report in the literature of EHL with DBE-ERCP without DPCS but supported only by enteroscopy view. This is also the first case reported in a liver transplant recipient. Finally, we describe a cost-effective strategy to preserve the EHL probe from early exhaustion.

Endoscopy_UCTN_Code_TTT_1AR_2AH

## Notes

Endoscopy E-Videos

https://eref.thieme.de/e-videos
*E-Videos* is an open access online section of the journal *Endoscopy* , reporting on interesting cases and new techniques in gastroenterological endoscopy. All papers include a high-quality video and are published with a Creative Commons CC-BY license. Endoscopy E-Videos qualify for HINARI discounts and waivers and eligibility is automatically checked during the submission process. We grant 100% waivers to articles whose corresponding authors are based in Group A countries and 50% waivers to those who are based in Group B countries as classified by Research4Life (see: https://www.research4life.org/access/eligibility/ ). This section has its own submission website at https://mc.manuscriptcentral.com/e-videos
